# Dietary magnesium intake and the risk of cardiovascular disease, type 2 diabetes, and all-cause mortality: a dose–response meta-analysis of prospective cohort studies

**DOI:** 10.1186/s12916-016-0742-z

**Published:** 2016-12-08

**Authors:** Xuexian Fang, Kai Wang, Dan Han, Xuyan He, Jiayu Wei, Lu Zhao, Mustapha Umar Imam, Zhiguang Ping, Yusheng Li, Yuming Xu, Junxia Min, Fudi Wang

**Affiliations:** 1 Department of Nutrition, Nutrition Discovery Innovation Center, Institute of Nutrition and Food Safety, Collaborative Innovation Center for Diagnosis and Treatment of Infectious Diseases, Beijing Advanced Innovation Center for Food Nutrition and Human Health, School of Public Health, School of Medicine, Zhejiang University, Hangzhou, China; 2 The First Affiliated Hospital, Institute of Translational Medicine, School of Medicine, Zhejiang University, Hangzhou, China; 3 Precision Nutrition Innovation Center, College of Public Health, Zhengzhou University, Zhengzhou, China; 4 Department of Neurology, The First Affiliated Hospital of Zhengzhou University, Zhengzhou, China

**Keywords:** Magnesium, Cardiovascular disease, Type 2 diabetes, All-cause mortality, Meta-analysis

## Abstract

**Background:**

Although studies have examined the association between dietary magnesium intake and health outcome, the results are inconclusive. Here, we conducted a dose–response meta-analysis of prospective cohort studies in order to investigate the correlation between magnesium intake and the risk of cardiovascular disease (CVD), type 2 diabetes (T2D), and all-cause mortality.

**Methods:**

PubMed, EMBASE, and Web of Science were searched for articles that contained risk estimates for the outcomes of interest and were published through May 31, 2016. The pooled results were analyzed using a random-effects model.

**Results:**

Forty prospective cohort studies totaling more than 1 million participants were included in the analysis. During the follow-up periods (ranging from 4 to 30 years), 7678 cases of CVD, 6845 cases of coronary heart disease (CHD), 701 cases of heart failure, 14,755 cases of stroke, 26,299 cases of T2D, and 10,983 deaths were reported. No significant association was observed between increasing dietary magnesium intake (per 100 mg/day increment) and the risk of total CVD (RR: 0.99; 95% CI, 0.88–1.10) or CHD (RR: 0.92; 95% CI, 0.85–1.01). However, the same incremental increase in magnesium intake was associated with a 22% reduction in the risk of heart failure (RR: 0.78; 95% CI, 0.69–0.89) and a 7% reduction in the risk of stroke (RR: 0.93; 95% CI, 0.89–0.97). Moreover, the summary relative risks of T2D and mortality per 100 mg/day increment in magnesium intake were 0.81 (95% CI, 0.77–0.86) and 0.90 (95% CI, 0.81–0.99), respectively.

**Conclusions:**

Increasing dietary magnesium intake is associated with a reduced risk of stroke, heart failure, diabetes, and all-cause mortality, but not CHD or total CVD. These findings support the notion that increasing dietary magnesium might provide health benefits.

**Electronic supplementary material:**

The online version of this article (doi:10.1186/s12916-016-0742-z) contains supplementary material, which is available to authorized users.

## Background

Magnesium is the eighth most common element in our planet’s crust and a biologically active mineral essential for life. All cells require magnesium, which acts as a critical cofactor for hundreds of enzymes involved in glucose metabolism, protein production, and nucleic acid synthesis [1]. Due to the daily loss of magnesium in feces, urine, and sweat [2], humans require magnesium intake (for example, by consuming magnesium-rich foods such as whole grains, green leafy vegetables, and nuts) in order to maintain normal magnesium levels [3, 4].

Despite the availability of many magnesium-rich foods, magnesium deficiency (i.e., hypomagnesemia, which is defined as a serum magnesium concentration < 0.74 mmol/L) is relatively common, with an estimated prevalence of 2.5–15% in the general population [5, 6]. The primary cause of hypomagnesemia is often insufficient dietary intake. Dietary magnesium is absorbed primarily by the small intestine via passive paracellular transport, which is driven by an electrochemical gradient and solvent drag [7, 8]. Interestingly, the intestinal absorption of magnesium is not directly proportional to magnesium intake, but depends primarily on the body’s magnesium status [9]. Peacock et al. once reported a correlation coefficient of 0.053 between baseline dietary and serum magnesium in a cohort study [10]. Even in developed countries such as the United States, many adults fail to meet the recommended daily intake of magnesium [11], despite the fact that epidemiology studies indicate that low levels of serum magnesium can increase the risk of a wide range of diseases, including chronic obstructive pulmonary disease [12], metabolic syndrome [13], type 2 diabetes (T2D) [14], Alzheimer’s disease [15], and cardiovascular disease (CVD) [16]. Therefore, understanding the relationship between dietary magnesium intake and overall health outcome is important for guiding public awareness and establishing clear dietary guidelines, thereby reducing the risk of magnesium deficiency-related diseases.

Previous meta-analyses suggest that the consumption of magnesium is associated with a reduced incidence of CVD and diabetes [17–19]. In recent years, a growing number of well-designed population-based studies focused on the relationship between magnesium and the risk of CVD, diabetes, and all-cause mortality, providing results that might be of great importance with respect to public health issues. However, these studies varied with respect to sample size, magnesium intake, and other characteristics, thereby contributing to inconsistencies within the literature [20, 21]. Therefore, we performed a comprehensive meta-analysis of all published prospective cohort studies in order to quantify the dose–response relationship between dietary magnesium intake and the risk of CVD, T2D, and all-cause mortality.

## Methods

This meta-analysis was designed, implemented, analyzed, and reported in accordance with the Meta-analysis of Observational Studies in Epidemiology (MOOSE) protocol [22].

### Search strategy

We systematically searched the databases PubMed, Embase, and Web of Science for prospective cohort studies published through May 31, 2016. The following keywords were used in the literature search: “magnesium” AND (“cardiovascular disease” OR “coronary heart disease” OR “myocardial infarction” OR “heart failure” OR “stroke” OR “cerebrovascular disease” OR “ischemic heart disease” OR “diabetes” OR “mortality” OR “death”) AND (“cohort” OR “prospective” OR “follow-up” OR “longitudinal” OR “population”). Our search was restricted to studies conducted in humans, and no restriction was imposed with respect to the language of the publications. The references cited within the retrieved relevant articles were also reviewed in order to identify additional studies.

### Study selection

Studies that satisfied the following four criteria were included in our meta-analysis: (1) prospective study design; (2) the exposure of interest was dietary intake of magnesium; (3) the outcome was CVD (including coronary heart disease/ischemic heart disease, and/or stroke), type 2 diabetes, and/or all-cause mortality; and (4) the authors reported risk estimates with 95% confidence intervals (95% CI). We excluded reviews, meta-analyses, retrospective studies, and published letters that lacked sufficient data. To ensure the correct identification of eligible studies, we used a two-step selection process [23]. First, two independent investigators (authors XF and KW) conducted an initial screening of all titles and/or abstracts; the full text of each potentially relevant article was then evaluated.

### Data extraction

Data were extracted using a standardized data collection form. Two investigators (authors XF and KW) independently extracted detailed information from each included article. Any discrepancies were resolved through group discussion with a third investigator (author FW). We extracted the following information: the first author of the publication, the year of publication, study location, study name (where applicable), the duration of follow-up, sex, sample size (the number of cases and/or participants), the method used to assess dietary intake (food-frequency questionnaire, 24-h recall, or other), the categories of magnesium intake, and the corresponding risk estimates with 95% CIs. We extracted the risk estimates with the most adjustment.

Quality assessment was performed in accordance with the Newcastle-Ottawa scale for non-randomized studies [24]. This scale assigns a maximum of 9 points to each study as follows: 4 points for the selection of participants and measurement of exposure, 2 points for comparability, and 3 points for assessment of outcomes and adequate follow-up. We regarded scores of 0–3, 4–6, and 7–9 as reflecting low, moderate, and high quality, respectively.

### Statistical analysis

Relative risk (RR) with the 95% CI was used as the common measure of association across studies; the hazard ratio and incidence rate ratio were considered to approximate RR. Studies that stratified the data by sex and/or stroke subgroup were treated as two separate reports. We used a random-effects model to calculate the summarized RRs and their corresponding 95% CIs for comparison between the highest and lowest levels of magnesium intake [25].

Due to the relatively wide range of definitions for the exposure categories in the included articles, we performed a dose-response analysis based on a 100 mg/day increase in magnesium intake, using the method recommended by Greenland and Longnecker and the publicly available Stata code written by Orsini et al*.* [26, 27]. The categories of magnesium intake, distributions of cases and person-years, and RRs and 95% CIs were extracted. If the number of cases and/or person-years was not available, variance-weighted least squares regression was used to achieve the pooled risk estimate [28, 29]. If neither median nor mean values were reported, we used the categorical midpoint. If the highest or lowest category was open-ended, the midpoint of the category was estimated by assuming that the width of the category was the same as the next adjacent category. In addition, we evaluated the non-linear association between dietary magnesium intake and risk of outcomes using restricted cubic splines, with three knots at the 10th, 50th, and 90th percentiles of the distribution [30]. A *P* value for curve linearity or non-linearity was calculated by testing the null hypothesis that the coefficient of the second spline is equal to zero.

Heterogeneity among the studies was estimated using the *I* ^*2*^ statistic, with values of 25%, 50%, and 75% representing low, moderate, and high degrees of heterogeneity, respectively [31, 32]. To explore the significance of the difference in RRs and the possible influence of residual confounding factors, we performed subgroup analyses and a meta-regression analysis on possible sources of heterogeneity, including sex, geographic location, and stroke subtype [33].

Publication bias was evaluated using contour-enhanced funnel plots, Egger’s linear regression test, and Begg’s rank correlation test, with significance set to *P* < 0.10 [34–36]. All statistical analyses were performed using Stata version 12. Except where noted otherwise, differences with a *P* value < 0.05 were considered significant.

## Results

### Study characteristics

**Fig. 1:**
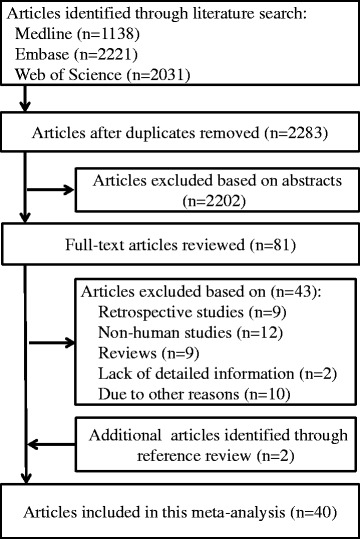
Flow-chart depicting the literature search and selection strategy

Figure 1 shows the study selection process and the results of our literature search. Using our search strategy (see Methods), we identified 1128 articles from PubMed, 2221 articles from Embase, and 2031 articles from Web of Science. After duplicates and studies that did not meet the inclusion criteria were excluded, 40 publications comprising 70 studies were ultimately included in our main analysis [20, 21, 37–74]. Because some studies contributed to more than one outcome, and because results stratified by sex and subtypes were treated separately, a total of 70 reports and/or datasets were included in the final meta-analysis. These prospective studies were published from 1999 through 2016, and the follow-up periods ranged from 4 to 30 years. Twenty-two studies were conducted in the United States, six in China, five in Japan, two in Sweden, and one each in the United Kingdom, Spain, Australia, Finland, and Germany. In total, we identified 7678 cases of CVD; 6845 cases of coronary heart disease (CHD); 701 cases of heart failure; 14,755 cases of stroke; 26,299 cases of T2D, and 10,983 cases of all-cause mortality. Dietary magnesium intake was assessed using a validated food frequency questionnaire in all studies except one. Study quality scores ranged from 7 to 9; the mean quality score was 8.2 (Additional file 1: Table S1). Detailed characteristics regarding the studies included in our analysis are summarized in Table 1.

**Table 1:** Characteristics of the included prospective cohort studies

Author, year	Location	Study name	Sex	Age range (years)	Follow-up (years)	Cases (cohort size)	Dietary assessment	Quality score
Adebamowo et al., 2015 [20]	US	Nurses’ Health Study (NHS)	F	30–55 in NHS I; 25–42 in NHS II	30 in NHS I; 22 in NHS II	3780 stroke cases (86,149 in NHS I; 94,715 in NHS II)	Validated FFQ	8
Adebamowo et al., 2015 [37]	US	Health Professionals Follow-up Study (HPFS)	M	40–75	24	1547 stroke cases (42,669)	Validated FFQ	9
Al-Delaimy et al., 2004 [38]	US	HPFS	M	40–75	12	1449 CHD cases (39,633)	Validated FFQ	9
Ascherio et al., 1998 [39]	US	HPFS	M	40–75	8	328 stroke cases (43,738)	Validated FFQ	9
Bain et al., 2015 [40]	UK	European Prospective Investigation into Cancer (EPIC)-Norfolk cohort	M/F	40–75	5.8	928 stroke cases (25,639)	7-day dietary recall	9
Chiuve et al., 2011 [41]	US	NHS	F	30–55	26	505 sudden cardiac deaths (88,375)	Validated FFQ	9
Chiuve et al., 2013 [42]	US	NHS	F	30–55	28	3614 CHD cases (86,323)	Validated FFQ	8
Dai et al., 2013 [21]	China	Shanghai Women’s Health Study (SWHS) and Shanghai Men’s Health Study (SMHS)	M/F	40–70 in SWHS; 40–74 in SMHS	NA	6224 total deaths, 1947 CVD deaths, 906 CHD deaths, 1041 stroke deaths (136,442)	Validated FFQ	9
de Oliveira Otto et al., 2012 [43]	US	Multi-Ethnic Study of Atherosclerosis. Participants (MESA)	M/F	45–84	6.2	279 CVD case, 399 T2D cases (6814)	Validated FFQ	7
Guasch-Ferré et al., 2014 [44]	Spain	Prevención con Dieta Mediterránea (PREDIMED)	M/F	55–80	4.8	323 total deaths, 81 CVD deaths, 277 CVD cases (7216)	Validated FFQ	7
Hata et al., 2013 [45]	Japan	The Hisayama Study	M/F	40–79	15.6	417 T2D cases (1999)	Validated FFQ	9
Hodge et al., 2004 [46]	Australia	Melbourne Collaborative Cohort Study	M/F	40–69	4	365 T2D cases (31,641)	Validated FFQ	8
Hopping et al., 2010 [47]	Hawaii, US	Multiethnic Cohort (MEC)	M/F	45–75	14	8587 T2D cases (75,512)	Validated FFQ	8
Hruby et al., 2014 [48]	US	Framingham Heart Study (FHS) Offspring cohort	M/F	26–81	7	179 T2D cases (2582)	Validated FFQ	7
Huang et al., 2015 [49]	Taiwan, China	Nutrition and Health Survey in Taiwan	M/F	65–97	9	475 total deaths, 124 CVD deaths, 231 diabetes cases (1400)	24-h dietary recall and validated FFQ	7
Iso et al., 1999 [50]	US	NHS	F	34–59	14	690 stroke cases (85,764)	Validated FFQ	8
Kaluza et al., 2010 [51]	Sweden	Cohort of Swedish Men	M	45–79	10	2358 total deaths, 819 CVD deaths (23,366)	Validated FFQ	8
Kao et al., 1999 [52]	US	Atherosclerosis Risk in Communities (ARIC) Study	M/F	45–64	6	1106 T2D cases (12,128)	12-h dietary recall	8
Kim et al., 2010 [53]	US	Coronary Artery Risk Development in Young Adults Study	M/F	18–30	20	330 diabetes cases (4497)	Validated FFQ	9
Kirii et al., 2010 [54]	Japan	Japan Collaborative Cohort Study (JACC)	M/F	40–65	5	459 diabetes cases (11,592)	Validated FFQ	7
Konishi et al., 2015 [55]	Japan	The Takayama Study	M/F	>35	10	438 diabetes case (13,525)	Validated FFQ	8
Larsson et al., 2008 [56]	Finland	Alpha-Tocopherol, Beta-Carotene Cancer Prevention Study	M	50-69	13.6	2702 stroke cases (26,556)	Validated FFQ	9
Larsson et al., 2011 [57]	Sweden	Swedish Mammography Cohort	F	49-83	10.4	1680 stroke cases (34,670)	Validated FFQ	7
Levitan et al., 2013 [58]	US	Women’s Health Initiative	F	50-79	4.6	1433 total deaths (161,808)	Validated FFQ	7
Liao et al., 1998 [59]	US	ARIC	M/F	45-64	4-7	319 CHD cases (13,922)	Validated FFQ	8
Lin et al., 2013 [60]	Taiwan, China	Cardiovascular Disease Risk Factor Two-township Study (CVDFACTS)	M/F	>18	12	123 stroke cases (2061)	Validated FFQ	8
Lopez-Ridaura et al., 2004 [61]	US	NHS and HPFS	M/F	30-55 in NHS; 40-75 in HPFS	18 in NHS; 12 in HPFS	4085 T2D cases in NHS (85,060); 1333 T2D cases in HPFS (42,872)	Validated FFQ	9
Meyer et al., 2000 [62]	US	Iowa Women’s Health Study	F	55-69	6	1141 T2D cases (35,988)	Validated FFQ	9
Nanri et al., 2010 [63]	Japan	Japan Public Health Center-based Prospective Study	M/F	45-75	5	1114 T2D cases (59,791)	Validated FFQ	8
Ohira et al., 2009 [64]	US	ARIC	M/F	45-64	15	577 ischemic stroke cases (14,221)	Validated FFQ	9
Schulze et al., 2007 [65]	Germany	EPIC-Potsdam study	M/F	35-65	7	844 T2D cases (25,067)	Validated FFQ	9
Song et al., 2004 [66]	US	Women’s Health Study (WHS)	F	>45	6	918 T2D cases (39,345)	Validated FFQ	9
Song et al., 2005 [67]	US	WHS	F	>45	10	1037 CVD cases (39,876)	Validated FFQ	9
Tao et al., 2016 [68]	US	Western New York Exposures and Breast Cancer Study	F	35-79	7.3	170 all-cause deaths (1170)	Validated FFQ	8
Taveira et al., 2016 [69]	US	Jackson Heart Study	M/F	55-74	5	270 heart failure hospitalizations (4916)	Validated FFQ	9
van Dam et al., 2006 [70]	US	Black Women’s Health Study	F	21-69	8	1964 T2D cases (41,186)	Validated FFQ	9
Villegas et al., 2009 [71]	China	Shanghai Women’s Health Study	F	40-70	6.9	2270 T2D cases (64,191)	Validated FFQ	9
Weng et al., 2008 [72]	Taiwan, China	CVDFACTS	M/F	>40	10.6	132 ischemic stroke (1772)	Validated FFQ	8
Weng et al., 2012 [73]	Taiwan, China	CVDFACTS	M/F	>30	4.6	141 T2D cases (1604)	Validated FFQ	7
Zhang et al., 2012 [74]	Japan	JACC	M/F	40-79	14.7	2690 CVD deaths, 1227 stroke deaths, 557 CHD deaths, 431 heart failure deaths (58,615)	Validated FFQ	8

*FFQ* Food-frequency questionnaire, *NA* Not available, *T2D* type 2 diabetes, *CVD* cardiovascular disease, *CHD* coronary heart disease

### Dietary magnesium intake and the risk of CVD

**Fig. 2:**
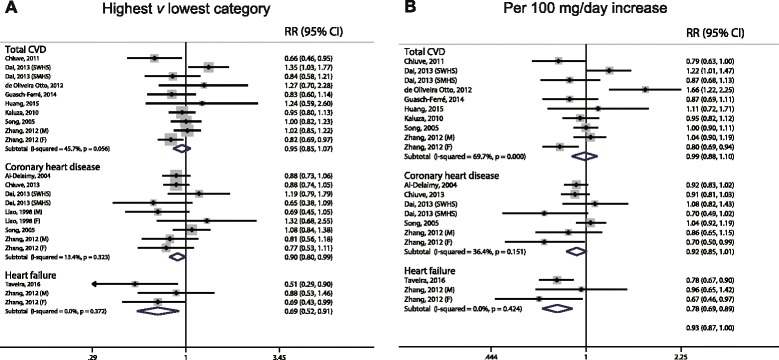
Forest plots of total cardiovascular disease, coronary heart disease, and heart failure for the highest versus lowest categories of dietary magnesium intake (**a**) and per 100 mg/day increase in dietary magnesium intake (**b**).

Ten independent reports from eight studies investigated the association between dietary magnesium intake and the risk of CVD. The pooled results suggest that magnesium intake is not significantly associated with CVD, which was suggested both by the highest category versus lowest category (RR: 0.95; 95% CI, 0.85–1.07) and by the per 100 mg/day increase (RR: 0.99, 95% CI, 0.88–1.10) (Fig. 2); in addition, we found evidence of between-study heterogeneity (*I* ^*2*^ = 45.7% and 69.7%, respectively). We also found no evidence of a non-linear association between dietary magnesium and CVD risk (*P* = 0.097 for non-linearity; see Fig. 6a).

Nine reports from six studies were included in our analysis of the association between magnesium intake and CHD. We found that the highest category of dietary magnesium was associated with a 10% lower risk of CHD compared to the lowest category of dietary magnesium (RR: 0.90; 95% CI, 0.80–0.99; Fig. 2), with low heterogeneity (*I* ^*2*^ = 13.4%). One study was not included in the dose–response analysis because it did not report specific data regarding the level of magnesium intake [59]. When we pooled the data from the remaining five studies, we found that a 100 mg/day increase in dietary magnesium intake was not significantly associated with CHD risk (RR: 0.92; 95% CI, 0.85–1.01; Fig. 2). However, a significant non-linear association was observed using the restricted cubic splines model (*P* < 0.05 for non-linearity; Fig. 6b).

At the time of our latest search, only three published datasets from two independent cohorts reported the association between magnesium intake and heart failure. We pooled these results and found strong inverse correlations for both the highest category versus lowest category (RR: 0.69; 95% CI, 0.52–0.91) and for a per 100 mg/day increase (RR: 0.78, 95% CI, 0.69–0.89), with no heterogeneity. Because of the limited number of datasets, non-linear association was not investigated in this study.

### Dietary magnesium intake and the risk of stroke

**Fig. 3:**
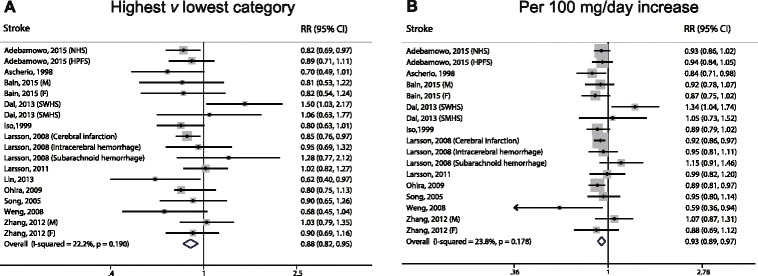
Forest plots of stroke for the highest versus lowest categories of dietary magnesium intake (**a**) and per 100 mg/day increase in dietary magnesium intake (**b**)

Fourteen prospective cohort studies estimated the risk of total stroke between the highest and lowest levels of magnesium intake; these results suggest a significant inverse correlation (RR: 0.88; 95% CI, 0.82–0.95; Fig. 3), with low heterogeneity (*I* ^*2*^ = 22.2%). After we excluded one study that was ineligible due to a lack of detailed magnesium categories [60], the dose–response analysis revealed that for each 100 mg/day increase in magnesium intake, the risk of stroke decreased by 7% (RR: 0.93; 95% CI, 0.89–0.97; Fig. 3), with low heterogeneity (*I* ^*2*^ = 23.8%). Accordingly, we also found a significant non-linear association (*P* < 0.001 for non-linearity; Fig. 6C).

### Dietary magnesium intake and the risk of type 2 diabetes

**Fig. 4:**
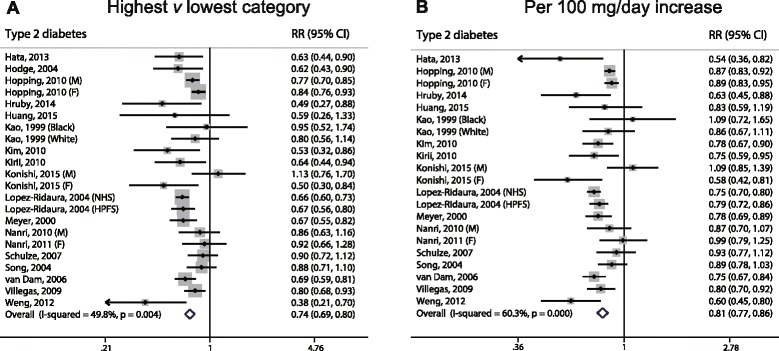
Forest plots of type 2 diabetes for the highest versus lowest categories of dietary magnesium intake (**a**) and per 100 mg/day increase of dietary magnesium intake (**b**)

The multivariable-adjusted RRs of T2D are shown in Fig. 4. When we compared the highest category of magnesium intake with the lowest category of magnesium intake, the pooled RR of T2D was 0.74 (95% CI, 0.69–0.80), with moderate heterogeneity (*I* ^*2*^ = 49.8%). When we examined the risk associated with a 100 mg/day increase in magnesium intake, the pooled RR was 0.81 (95% CI, 0.77–0.86), with relatively high heterogeneity (*I* ^*2*^ = 60.3%). We also found a significant non-linear dose–response relationship between magnesium intake and T2D (*P* < 0.001 for non-linearity; Fig. 6d).

### Dietary magnesium intake and all-cause mortality

**Fig. 5:**
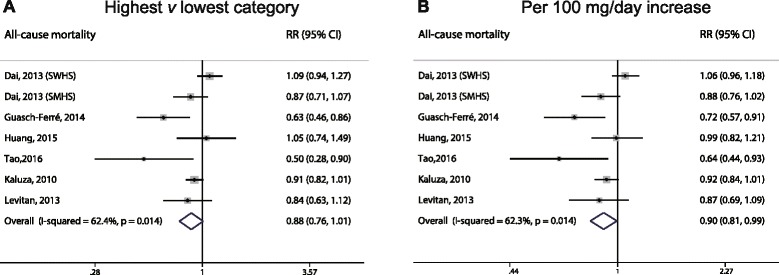
Forest plots of all-cause mortality for the highest versus lowest categories of dietary magnesium intake (**a**) and per 100 mg/day increase in dietary magnesium intake (**b**)

**Fig. 6:**
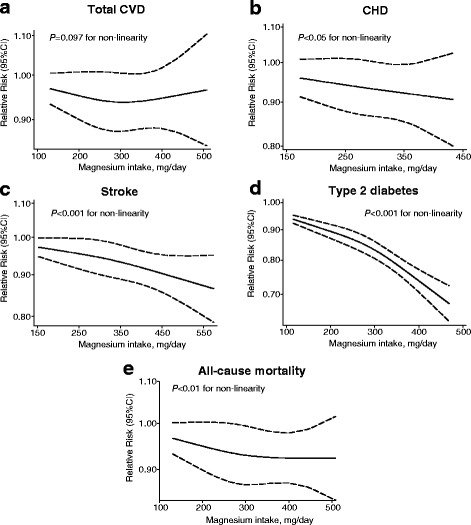
Dose–response analyses of the non-linear association between dietary magnesium intake and the risk of total cardiovascular disease (**a**), coronary heart disease (**b**), stroke (**c**), type 2 diabetes (**d**), and all-cause mortality (**e**)

With respect to all-cause mortality, the association with dietary magnesium intake was not statistically significant between the highest and lowest intake categories (RR: 0.88; 95% CI, 0.76–1.01; Fig. 5), with moderately high heterogeneity (*I* ^*2*^ = 62.4%). However, a dose–response analysis revealed that each 100 mg/day increase in dietary magnesium intake was associated with a 10% lower risk of mortality (RR: 0.90; 95% CI, 0.81–0.99; Fig. 5), with moderately high heterogeneity (*I* ^*2*^ = 62.3%). A cubic spline model revealed an inverse non-linear correlation between dietary magnesium intake and the risk of all-cause mortality (*P* < 0.01 for non-linearity; Fig. 6e).

### Subgroup analyses

Next, we performed subgroup analyses (with sex and study location as subgroups) in order to examine the stability of the primary results; these results are summarized in Table 2. The associations between dietary magnesium intake and the risks of CVD, T2D, and all-cause mortality were similar between our main analyses and our subgroup analyses, and meta-regression did not reveal any substantial change in the pooled relative risks. However, the inverse correlation remained significant only between increased magnesium intake and stroke incidence (RR: 0.92; 95% CI, 0.89–0.95), but not between increased magnesium intake and mortality (RR: 1.07; 95% CI, 0.90–1.28; *P* = 0.028 for meta-regression), and no study heterogeneity was observed (*I* ^*2*^ = 0%).

**Table 2:** Subgroup analyses (per 100 mg/day increase)

	N	RR (95% CI)	*I* ^*2*^ (%)	*P* value
Total cardiovascular disease				
Sex				
Male	3	0.98 (0.89–1.08)	0.0	0.959
Female	4	0.94 (0.73–1.13)	79.9	
Case				
Mortality	8	0.93 (0.82–1.05)	63.9	0.255
Incidence	3	1.10 (0.82–1.48)	82.7	
Location				
United States	3	1.07 (0.78–1.48)	86.2	0.606
Europe	2	0.93 (0.81–1.05)	0.0	
Asia	5	0.99 (0.83–1.17)	70.8	
Coronary heart disease				
Sex				
Male	3	0.89 (0.81–0.99)	3.5	0.409
Female	4	0.95 (0.83–1.09)	51.8	
Case				
Mortality	5	0.81 (0.69–0.95)	36.3	0.105
Incidence	3	0.97 (0.90–1.05)	12.7	
Location				
United States	3	0.95 (0.88–1.03)	27.6	0.439
Asia	4	0.84 (0.68–1.03)	42	
Stroke				
Subtype				
Ischemic	10	0.93 (0.88–0.98)	28.9	0.285
Hemorrhagic	9	0.97 (0.88–1.07)	29.0	
Sex				
Male	8	0.93 (0.89–0.98)	0.9	0.884
Female	7	0.94 (0.87–1.02)	37.2	
Case				
Mortality	4	1.07 (0.90–1.28)	45.0	0.028
Incidence	13	0.92 (0.89–0.95)	0.0	
Location				
United states	6	0.91 (0.87–0.95)	0.0	0.140
Europe	6	0.93 (0.89–0.98)	0.0	
Asia	5	1.00 (0.80–1.23)	63.5	
Type 2 diabetes				
Sex				
Male	5	0.85 (0.79–0.93)	48.3	0.381
Female	9	0.81 (0.75–0.87)	65.8	
Location				
United States	11	0.81 (0.77–0.86)	63.2	0.968
Asia	9	0.79 (0.69–0.90)	62.4	
All-cause mortality				
Sex				
Male	2	0.91 (0.84–0.98)	0.0	0.745
Female	3	0.88 (0.68–1.14)	62.5	
Location				
United States	2	0.77 (0.58–1.04)	46.9	0.134
Europe	2	0.83 (0.66–1.06)	72.6	
Asia	3	0.98 (0.87–1.11)	51.5	

*RR* relative risk, *95% CI* 95 confidence interval (lower limit–upper limit)

### Publication bias

Visual inspection of funnel plots revealed no significant publication bias (Additional file 1: Figure S1 and Figure S2). In addition, both Egger’s linear regression test and Begg’s rank correlation test revealed little evidence of publication bias with respect to magnesium intake in relation to the risk of CVD, CHD, stroke, diabetes, or all-cause mortality (Additional file 1: Table S2). The assessment of publication bias was based on the fully adjusted model.

## Discussion

This systemic meta-analysis was based on 40 prospective cohort studies, with more than 1 million participants and 67,261 cases in nine countries. Thus, this meta-analysis provides the most up-to-date epidemiological evidence supporting the putative protective effect of magnesium intake against stroke, heart failure, diabetes, and all-cause mortality. A dose–response analysis revealed that a 100 mg/day increase in dietary magnesium intake is significantly associated with a 7%, 22%, 19%, and 10% decrease in the risk of stroke, heart failure, type 2 diabetes, and all-cause mortality, respectively. However, no clear association was found between magnesium intake and the risk of coronary heart disease or total cardiovascular disease, which may have been due – at least in part – to the relatively limited number of studies included in our analysis [75]. Further subgroup analyses did not reveal any significant effect of geographic location or sex on the respective correlations between magnesium intake and disease risk.

Magnesium plays an important role in maintaining human health. A typical adult contains a total of approximately 22–26 grams of magnesium, with 60% in skeletal tissue, and 39% and 1% located in intra- and extracellular regions, respectively [76]. Magnesium is essential to all living organisms, as it controls the function of many crucial enzymes, including those that utilize or synthesize ATP [77]. The recommended dietary allowance of magnesium is 350 mg/day for an average male adult and 300 mg/day for an average adult female, with an additional 150 mg/day during pregnancy and lactation [2]. However, despite these clearly established recommendations, magnesium deficiency remains a global public health problem. For example, diet surveys conducted in both Europe and the United States revealed that the daily intake of magnesium is generally lower than the recommended amounts [77]. Moreover, a major cause of magnesium deficiency can be attributed to an improperly balanced diet and/or impaired intestinal absorption [5, 78].

Compared to oral supplements and intravenous infusion, increasing magnesium intake through diet may only moderately increase one’s magnesium levels; however, increasing dietary magnesium intake is both safe and effective. For example, green leafy vegetables such as spinach provide magnesium through an abundance of chlorophyll molecules. Spices, nuts, beans, cocoa, and whole grains are also rich sources of magnesium [2]. Importantly, although these foods contain relatively high levels of magnesium, the daily requirement for magnesium is difficult to achieve through a single serving of any one food item. Therefore, consuming a wide variety of magnesium-rich foods will help ensure adequate daily intake of magnesium. Here, we focused our analysis on the association between dietary magnesium intake and the incidence of highly prevalent chronic diseases and all-cause mortality.

Larsson et al. [79] previously reported that dietary magnesium intake is inversely correlated with the risk of stroke, and many observational studies have confirmed that magnesium intake affects the risk of cardiovascular disease. However, these studies may have limited implications due to the lack of a non-linear analysis. In our study, we quantitatively investigated the associations between dietary magnesium intake and specific cardiovascular risks, including total CVD, CHD, heart failure, and stroke, by performing a dose–response meta-analysis. Notably, this is the first meta-analysis to investigate the effect of dietary magnesium intake on the risk of heart failure. Recently, Simental-Mendia et al. [80] systematically reviewed all published clinical trials and found beneficial effects of using oral magnesium supplements for 4 months or longer; specifically, insulin sensitivity and glucose control were improved in both diabetic and non-diabetic subjects [80]. Based on their analysis, we updated the most recent meta-analysis by Dong et al., who studied the effect of dietary magnesium on the risk of diabetes [19]. To the best of our knowledge, our study is the first quantitative meta-analysis to investigate the dose–response relationship between dietary magnesium intake and all-cause mortality.

Given the lack of large, randomized intervention trials to increase magnesium intake in order to prevent CVD and/or T2D, our comprehensive analysis of the most up-to-date prospective studies provides strong epidemiological evidence of the effect of dietary magnesium on CVD, T2D, and all-cause mortality. The strength of our meta-analysis lies in three aspects. First, we included all available prospective cohort studies with high quality scores, large sample sizes, and long-term follow-up data. According to the Newcastle-Ottawa scale, the average quality of the included studies was high. Second, the statistical power of our quantitative assessment was greatly increased by the large number of well-recorded cases. Finally, in addition to comparing the highest and lowest categories of magnesium intake, we also performed both linear and non-linear dose–response analyses.

On the other hand, our study has several limitations that warrant discussion. First, given the observational nature of the included studies, we cannot exclude the possibility of residual confounding, even in the fully adjusted models. Although a wide range of potential confounders, including demographics and lifestyle factors, were adjusted for in the primary studies, dietary factors may not have been considered to a sufficient degree. Therefore, we cannot exclude the possibility that other nutrients and/or dietary components correlated with dietary magnesium may have been responsible, either partially or entirely, for the observed associations. Moreover, the majority of dietary data were collected using a food frequency questionnaire; although such a questionnaire can adequately characterize dietary patterns, it can be limited in terms of describing the intake of individual nutrients. For example, Ward et al. [81] compared the data obtained from a food frequency questionnaire with diet diary data in a nested case-control study and found that food frequency questionnaires may not sufficiently capture heterogeneity within a single population; however, they can be appropriate in pooled analyses in which a wider range of intakes are collated [81]. Moreover, measurement error might occur in dietary assessment, which would likely bias true associations towards a null association [82]. In addition, some participants may have changed their diet but may not have updated their information during the follow-up period. A second limitation is that we may have overlooked some studies and/or missed unpublished reports, although every effort was made to contact authors in order to obtain unpublished risk estimates. Third, significant heterogeneity may exist with respect to our meta-analyses between magnesium intake and the relative risks of total CVD, diabetes, and/or all-cause mortality. Although we performed extensive subgroup analyses using meta-regression, the sources of heterogeneity remain unclear.

## Conclusions

In conclusion, we observed significant inverse correlations between dietary magnesium intake and the risk of stroke, heart failure, diabetes, and all-cause mortality; in contrast, we found no correlation between dietary magnesium intake and the risk of coronary heart disease or total cardiovascular disease. Our findings underscore the notion that increasing the consumption of magnesium-rich foods may be beneficial to overall health. In the future, large prospective randomized controlled trials should help identify the putative causal role that magnesium plays in reducing the incidence of these diseases.

## Additional file

Additional file 1: Table S1. Quality assessment and references of all included prospective cohort studies. **Table S2.** Publication bias measured by Begg’s and Egger’s test. **Figure S1.** Funnel plots for studies of the association between the highest *vs.* lowest category in dietary magnesium intake and risk of total CVD (A), CHD (B), stroke (C), type 2 diabetes (D), and all-cause mortality (E). **Figure S2.** Funnel plots for studies of the association between per 100 mg/day increase in dietary magnesium intake and risk of total CVD (A), CHD (B), stroke (C), type 2 diabetes (D), and all-cause mortality (E). (PDF 1552 kb)
