# Supplementary material for: Dietary magnesium intake and the risk of cardiovascular disease, type 2 diabetes, and all-cause mortality: a dose–response meta-analysis of prospective cohort studies
Source: BMC Med. 2016 Dec 8;14:210. doi: 10.1186/s12916-016-0742-z (PMC5143460; doi:10.1186/s12916-016-0742-z)

ONLINE SUPPLEMENTAL MATERIAL

Dietary magnesium intake and risk of cardiovascular disease, type 2 diabetes, and all-cause mortality: A dose-response meta-analysis of prospective cohort studies

Xuexian Fang, Kai Wang, Dan Han, Xuyan He, Jiayu Wei, Lu Zhao,
Mustapha Umar Imam, Zhiguang Ping, Junxia Min, Fudi Wang

Contents:

Table S1 Quality assessment and references of all included prospective cohort studies

Table S2 Publication bias measured by Begg's and Egger's test

Figure S1 Funnel plots for studies of the association between the highest *vs.* lowest category in dietary magnesium intake and risk of total CVD (A), CHD (B), stroke (C), type 2 diabetes (D), and all-cause mortality (E)

Figure S2 Funnel plots for studies of the association between per 100 mg/day increase in dietary magnesium intake and risk of total CVD (A), CHD (B), stroke (C), type 2 diabetes (D), and all-cause mortality (E)

Table S1 Quality assessment and references of all included prospective cohort studies

Author, year	Selection				Comparability	Outcome			Overall quality
	Representative of cases	Selection of controls	Exposure ascertainment	No history of disease	Comparable on confounders	Outcome assessment	Adequate follow-up time (> 5 years)	Follow-up rate (> 80%)	
Adebamowo *et al*，2015(1)	1	1	1	1	2	1	1	0	8
Adebamowo *et al*，2015(2)	1	1	1	1	1	1	1	1	9
Al-Delaimy *et al*, 2004(3)	1	1	1	1	2	1	1	1	9
Ascherio *et al*, 1998(4)	1	1	1	1	2	1	1	1	9
Bain *et al*, 2015(5)	1	1	1	1	2	1	1	1	9
Chiuve *et al*, 2011(6)	1	1	1	1	2	1	1	1	9
Chiuve *et al*, 2013(7)	1	1	1	1	2	1	1	0	8
Dai *et al*, 2013(8)	1	1	1	1	2	1	1	1	9
de Oliveira Otto *et al*, 2012(9)	1	1	1	1	2	1	0	0	7
Guasch-Ferré *et al*, 2014(10)	1	1	1	1	2	1	0	0	7
Hata *et al*, 2013(11)	1	1	1	1	2	1	1	1	9
Hodge *et al*, 2004(12)	1	1	1	0	2	1	0	1	7

Hopping *et al*, 2010(13)	1	1	1	1	2	1	1	0	8
Hruby *et al*, 2014(14)	1	1	1	0	2	1	1	0	7
Huang *et al*, 2015(15)	1	1	1	0	2	1	1	0	7
Iso *et al*, 1999(16)	1	1	1	0	2	1	1	1	8
Kaluza *et al*, 2010(17)	1	1	1	1	2	1	1	0	8
Kao *et al*, 1999(18)	1	1	1	1	2	1	1	0	8
Kim *et al*, 2010(19)	1	1	1	1	2	1	1	1	9
Kirii *et al*, 2010(20)	1	1	1	1	2	1	0	0	7
Konishi *et al*, 2015(21)	1	1	1	1	2	1	1	0	8
Larsson *et al*, 2008(22)	1	1	1	1	2	1	1	1	9
Larsson *et al*, 2011(23)	1	1	1	0	2	1	1	0	7

Levitan *et al*, 2013(24)	1	1	1	0	2	1	0	0	7
Liao *et al*, 1998(25)	1	1	1	0	2	1	1	1	8
Lin *et al*, 2013(26)	1	1	1	1	2	1	1	0	8
Lopez-Ridaura *et al*, 2004(27)	1	1	1	1	2	1	1	1	9
Meyer *et al*, 2000(28)	1	1	1	1	2	1	1	1	9
Nanri *et al*, 2010(29)	1	1	1	1	2	1	0	1	8
Ohira *et al*, 2009(30)	1	1	1	1	2	1	1	1	9
Schulze *et al*, 2007(31)	1	1	1	1	2	1	1	1	9
Song *et al*, 2004(32)	1	1	1	1	2	1	1	1	9
Song *et al*, 2005(33)	1	1	1	1	2	1	1	1	9
Tao *et al*, 2016(34)	1	1	1	0	2	1	1	1	8

Taveira *et al*, 2016(35)	1	1	1	1	2	1	1	1	9
van Dam *et al*, 2006(36)	1	1	1	1	2	1	1	1	9
Villegas *et al*, 2009(37)	1	1	1	1	2	1	1	1	9
Weng *et al*, 2008(38)	1	1	1	1	2	1	1	0	8
Weng *et al*, 2012(39)	1	1	1	1	2	1	0	0	7
Zhang *et al*, 2012(40)	1	1	1	1	2	1	1	0	8

Average: 8.2

1. Adebamowo SN, Spiegelman D, Willett WC, Rexrode KM. Association between intakes of magnesium, potassium, and calcium and risk of stroke: 2 cohorts of US women and updated meta-analyses. Am J Clin Nutr 2015; 101: 1269-77.
2. Adebamowo SN, Spiegelman D, Flint AJ, Willett WC, Rexrode KM. Intakes of magnesium, potassium, and calcium and the risk of stroke among men. Int J Stroke 2015; 10: 1093-100.
3. Al-Delaimy WK, Rimm EB, Willett WC, Stampfer MJ, Hu FB. Magnesium intake and risk of coronary heart disease among men. J Am Coll Nutr 2004; 23: 63-70.
4. Ascherio A, Rimm EB, Hernan MA, Giovannucci EL, Kawachi I, Stampfer MJ, Willett WC. Intake of potassium, magnesium, calcium, and fiber and risk of stroke among US men. Circulation 1998; 98: 1198-204.
5. Bain LK, Myint PK, Jennings A, Lentjes MA, Luben RN, Khaw KT, Wareham NJ, Welch AA. The relationship between dietary magnesium intake, stroke

and its major risk factors, blood pressure and cholesterol, in the EPIC-Norfolk cohort. Int J Cardiol 2015; 196: 108-14.
6. Chiuve SE, Korngold EC, Januzzi JL, Jr., Gantzer ML, Albert CM. Plasma and dietary magnesium and risk of sudden cardiac death in women. Am J Clin Nutr 2011; 93: 253-60.
7. Chiuve SE, Sun Q, Curhan GC, Taylor EN, Spiegelman D, Willett WC, Manson JE, Rexrode KM, Albert CM. Dietary and plasma magnesium and risk of coronary heart disease among women. J Am Heart Assoc 2013; 2: e000114.
8. Dai Q, Shu XO, Deng X, Xiang YB, Li H, Yang G, Shrubsole MJ, Ji B, Cai H, Chow WH et al. Modifying effect of calcium/magnesium intake ratio and mortality: a population-based cohort study. BMJ Open 2013; 3.
9. de Oliveira Otto MC, Alonso A, Lee DH, Delclos GL, Bertoni AG, Jiang R, Lima JA, Symanski E, Jacobs DR, Jr., Nettleton JA. Dietary intakes of zinc and heme iron from red meat, but not from other sources, are associated with greater risk of metabolic syndrome and cardiovascular disease. J Nutr 2012; 142: 526-33.
10. Guasch-Ferre M, Bullo M, Estruch R, Corella D, Martinez-Gonzalez MA, Ros E, Covas M, Aros F, Gomez-Gracia E, Fiol M et al. Dietary magnesium intake is inversely associated with mortality in adults at high cardiovascular disease risk. J Nutr 2014; 144: 55-60.
11. Hata A, Doi Y, Ninomiya T, Mukai N, Hirakawa Y, Hata J, Ozawa M, Uchida K, Shirota T, Kitazono T et al. Magnesium intake decreases Type 2 diabetes risk through the improvement of insulin resistance and inflammation: the Hisayama Study. Diabet Med 2013; 30: 1487-94.
12. Hodge AM, English DR, O'Dea K, Giles GG. Glycemic index and dietary fiber and the risk of type 2 diabetes. Diabetes Care 2004; 27: 2701-6.
13. Hopping BN, Erber E, Grandinetti A, Verheus M, Kolonel LN, Maskarinec G. Dietary fiber, magnesium, and glycemic load alter risk of type 2 diabetes in a multiethnic cohort in Hawaii. J Nutr 2010; 140: 68-74.
14. Hruby A, Meigs JB, O'Donnell CJ, Jacques PF, McKeown NM. Higher magnesium intake reduces risk of impaired glucose and insulin metabolism and progression from prediabetes to diabetes in middle-aged americans. Diabetes Care 2014; 37: 419-27.
15. Huang YC, Wahlqvist ML, Kao MD, Wang JL, Lee MS. Optimal Dietary and Plasma Magnesium Statuses Depend on Dietary Quality for a Reduction in the Risk of All-Cause Mortality in Older Adults. Nutrients 2015; 7: 5664-83.
16. Iso H, Stampfer MJ, Manson JE, Rexrode K, Hennekens CH, Colditz GA, Speizer FE, Willett WC. Prospective study of calcium, potassium, and magnesium intake and risk of stroke in women. Stroke 1999; 30: 1772-9.
17. Kaluza J, Orsini N, Levitan EB, Brzozowska A, Roszkowski W, Wolk A. Dietary calcium and magnesium intake and mortality: a prospective study of men. Am J Epidemiol 2010; 171: 801-7.
18. Kao WH, Folsom AR, Nieto FJ, Mo JP, Watson RL, Brancati FL. Serum and dietary magnesium and the risk for type 2 diabetes mellitus: the Atherosclerosis Risk in Communities Study. Arch Intern Med 1999; 159: 2151-9.
19. Kim DJ, Xun P, Liu K, Loria C, Yokota K, Jacobs DR, Jr., He K. Magnesium intake in relation to systemic inflammation, insulin resistance, and the incidence of diabetes. Diabetes Care 2010; 33: 2604-10.

20. Kirii K, Iso H, Date C, Fukui M, Tamakoshi A, Group JS. Magnesium intake and risk of self-reported type 2 diabetes among Japanese. J Am Coll Nutr 2010; 29: 99-106.
21. Konishi K, Wada K, Tamura T, Tsuji M, Kawachi T, Nagata C. Dietary magnesium intake and the risk of diabetes in the Japanese community: results from the Takayama study. Eur J Nutr 2015.
22. Larsson SC, Virtanen MJ, Mars M, Mannisto S, Pietinen P, Albanes D, Virtamo J. Magnesium, calcium, potassium, and sodium intakes and risk of stroke in male smokers. Arch Intern Med 2008; 168: 459-65.
23. Larsson SC, Virtamo J, Wolk A. Potassium, calcium, and magnesium intakes and risk of stroke in women. Am J Epidemiol 2011; 174: 35-43.
24. Levitan EB, Shikany JM, Ahmed A, Snetselaar LG, Martin LW, Curb JD, Lewis CE. Calcium, magnesium and potassium intake and mortality in women with heart failure: the Women's Health Initiative. Br J Nutr 2013; 110: 179-85.
25. Liao F, Folsom AR, Brancati FL. Is low magnesium concentration a risk factor for coronary heart disease? The Atherosclerosis Risk in Communities (ARIC) Study. Am Heart J 1998; 136: 480-90.
26. Lin PH, Yeh WT, Svetkey LP, Chuang SY, Chang YC, Wang C, Pan WH. Dietary intakes consistent with the DASH dietary pattern reduce blood pressure increase with age and risk for stroke in a Chinese population. Asia Pac J Clin Nutr 2013; 22: 482-91.
27. Lopez-Ridaura R, Willett WC, Rimm EB, Liu S, Stampfer MJ, Manson JE, Hu FB. Magnesium intake and risk of type 2 diabetes in men and women. Diabetes Care 2004; 27: 134-40.
28. Meyer KA, Kushi LH, Jacobs DR, Jr., Slavin J, Sellers TA, Folsom AR. Carbohydrates, dietary fiber, and incident type 2 diabetes in older women. Am J Clin Nutr 2000; 71: 921-30.
29. Nanri A, Mizoue T, Noda M, Takahashi Y, Kirii K, Inoue M, Tsugane S, Japan Public Health Center-based Prospective Study G. Magnesium intake and type II diabetes in Japanese men and women: the Japan Public Health Center-based Prospective Study. Eur J Clin Nutr 2010; 64: 1244-7.
30. Ohira T, Peacock JM, Iso H, Chambless LE, Rosamond WD, Folsom AR. Serum and dietary magnesium and risk of ischemic stroke: the Atherosclerosis Risk in Communities Study. Am J Epidemiol 2009; 169: 1437-44.
31. Schulze MB, Schulz M, Heidemann C, Schienkiewitz A, Hoffmann K, Boeing H. Fiber and magnesium intake and incidence of type 2 diabetes: a prospective study and meta-analysis. Arch Intern Med 2007; 167: 956-65.
32. Song Y, Manson JE, Buring JE, Liu S. Dietary magnesium intake in relation to plasma insulin levels and risk of type 2 diabetes in women. Diabetes Care 2004; 27: 59-65.
33. Song Y, Manson JE, Cook NR, Albert CM, Buring JE, Liu S. Dietary magnesium intake and risk of cardiovascular disease among women. Am J Cardiol 2005; 96: 1135-41.
34. Tao MH, Dai Q, Millen AE, Nie J, Edge SB, Trevisan M, Shields PG, Freudenheim JL. Associations of intakes of magnesium and calcium and survival among women with breast cancer: results from Western New York Exposures and Breast Cancer (WEB) Study. Am J Cancer Res 2016; 6: 105-13.

35. Taveira TH, Ouellette D, Gulum A, Choudhary G, Eaton CB, Liu S, Wu WC. Relation of Magnesium Intake With Cardiac Function and Heart Failure Hospitalizations in Black Adults: The Jackson Heart Study. Circ Heart Fail 2016; 9.
36. van Dam RM, Hu FB, Rosenberg L, Krishnan S, Palmer JR. Dietary calcium and magnesium, major food sources, and risk of type 2 diabetes in U.S. black women. Diabetes Care 2006; 29: 2238-43.
37. Villegas R, Gao YT, Dai Q, Yang G, Cai H, Li H, Zheng W, Shu XO. Dietary calcium and magnesium intakes and the risk of type 2 diabetes: the Shanghai Women's Health Study. Am J Clin Nutr 2009; 89: 1059-67.
38. Weng LC, Yeh WT, Bai CH, Chen HJ, Chuang SY, Chang HY, Lin BF, Chen KJ, Pan WH. Is ischemic stroke risk related to folate status or other nutrients correlated with folate intake? Stroke 2008; 39: 3152-8.
39. Weng LC, Lee NJ, Yeh WT, Ho LT, Pan WH. Lower intake of magnesium and dietary fiber increases the incidence of type 2 diabetes in Taiwanese. J Formos Med Assoc 2012; 111: 651-9.
40. Zhang W, Iso H, Ohira T, Date C, Tamakoshi A, Group JS. Associations of dietary magnesium intake with mortality from cardiovascular disease: the JACC study. Atherosclerosis 2012; 221: 587-95.

Table S2 Publication bias measured by Begg's and Egger's test

Outcomes	Highest *v* lowest category		Per 100 mg/day increase	
	Begg's test	Egger's tset	Begg's test	Egger's test
**Total CVD**	0.47	0.40	0.59	0.70
**Coronary heart disease**	0.60	0.90	0.13	0.30
**Stroke**	0.82	0.49	0.15	0.35
**Type 2 diabetes**	0.26	0.39	0.78	0.39
**All-cause mortality**	0.26	0.13	0.26	0.27

Figure S1

Funnel plots (highest *v* lowest)

A CVD

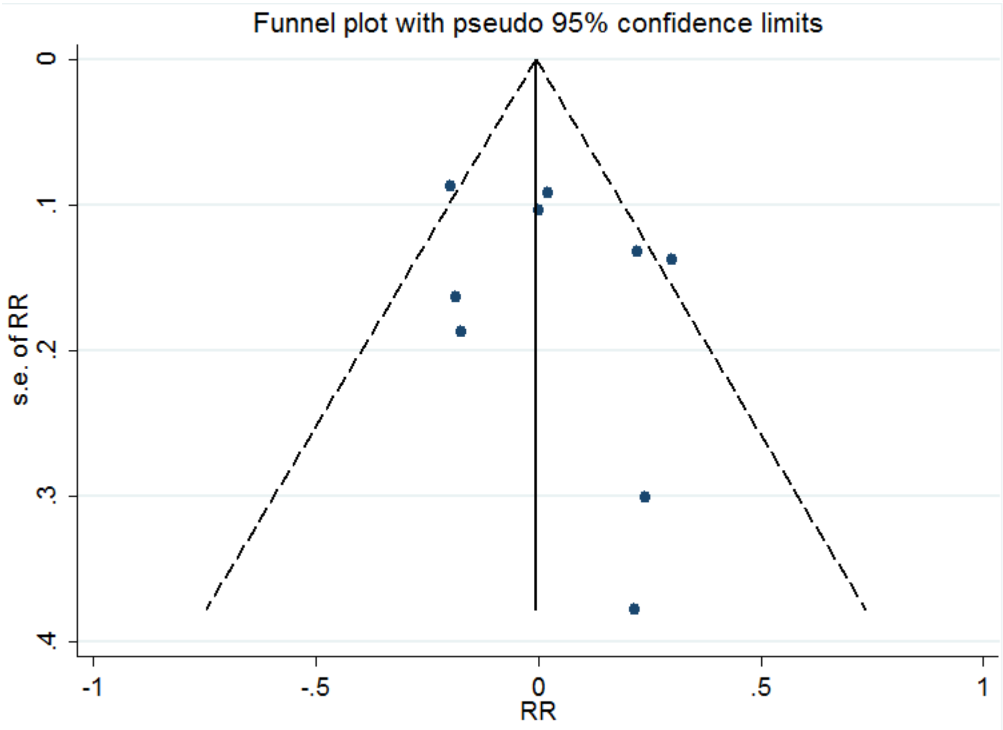

B CHD

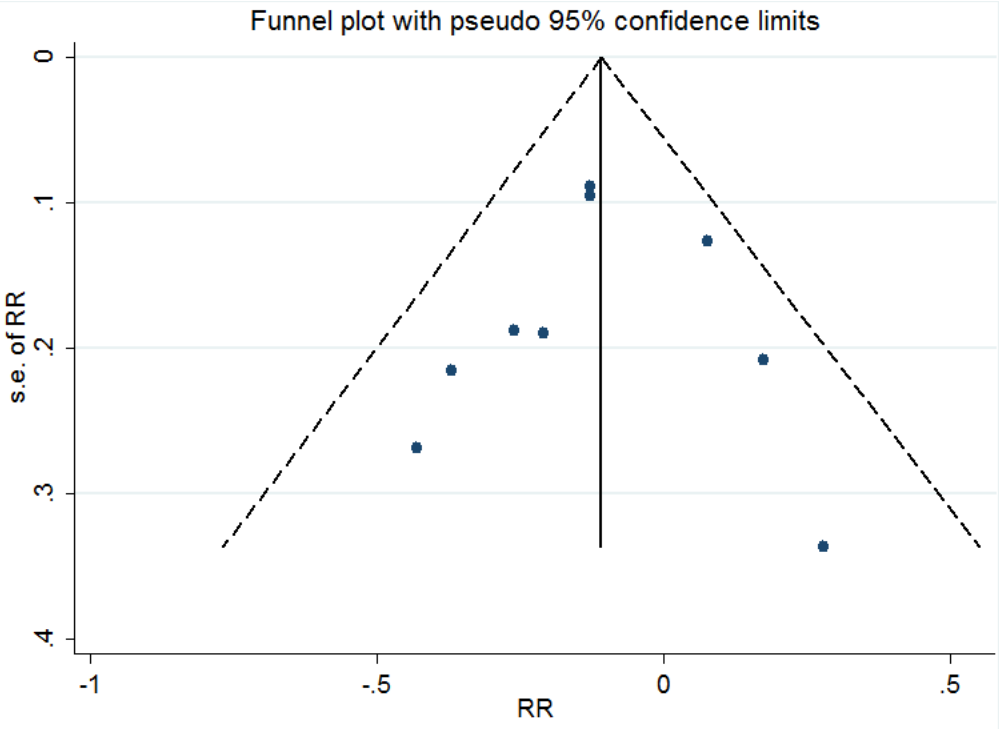

C Stroke

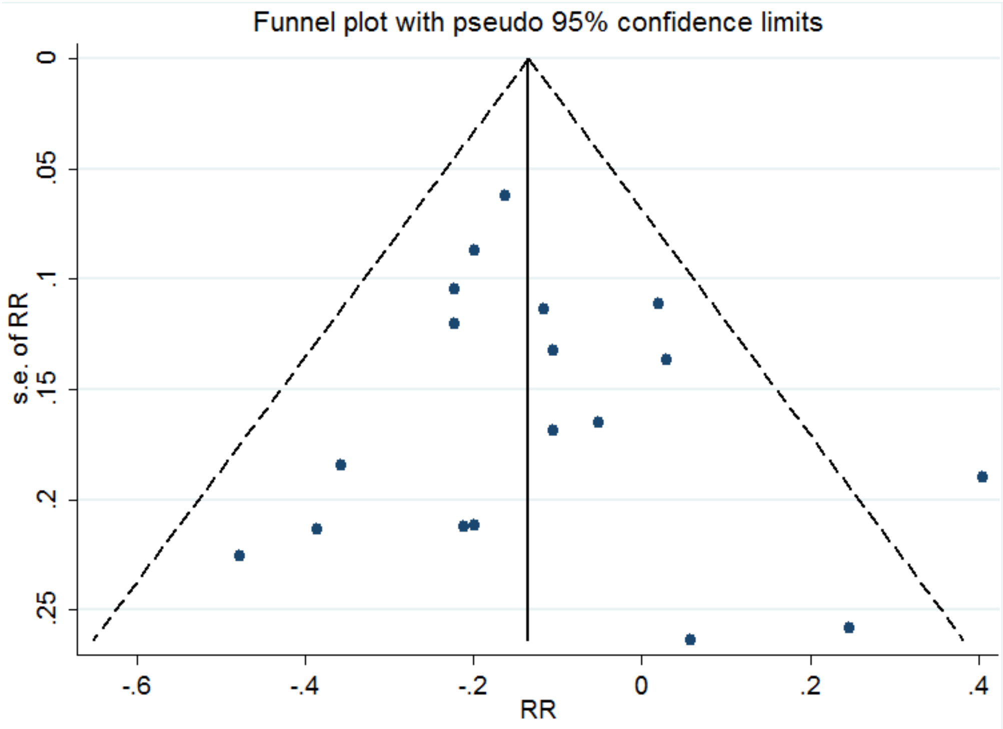

D T2DM

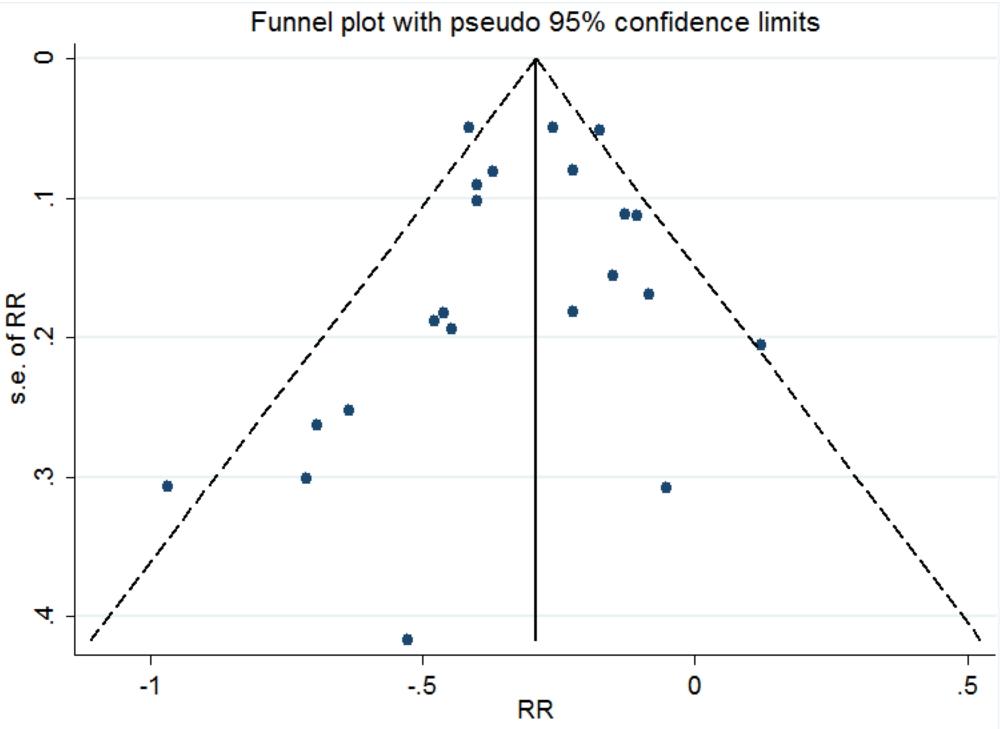

E All-cause mortality

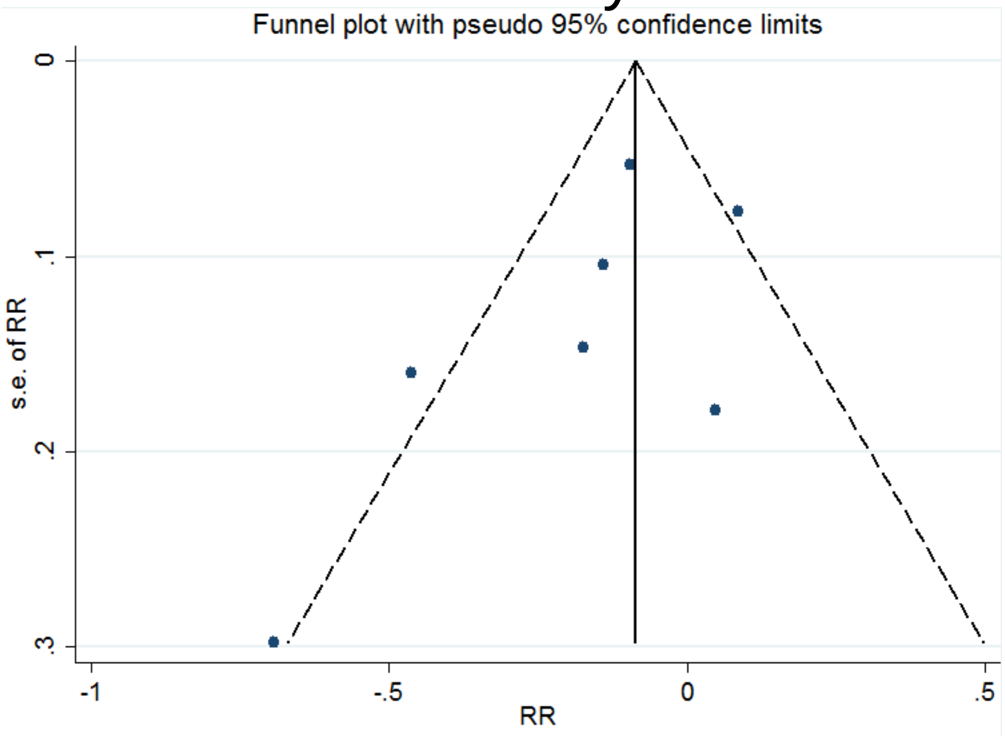

Figure S2

Funnel plots (Per 100 mg/day increase)

A CVD

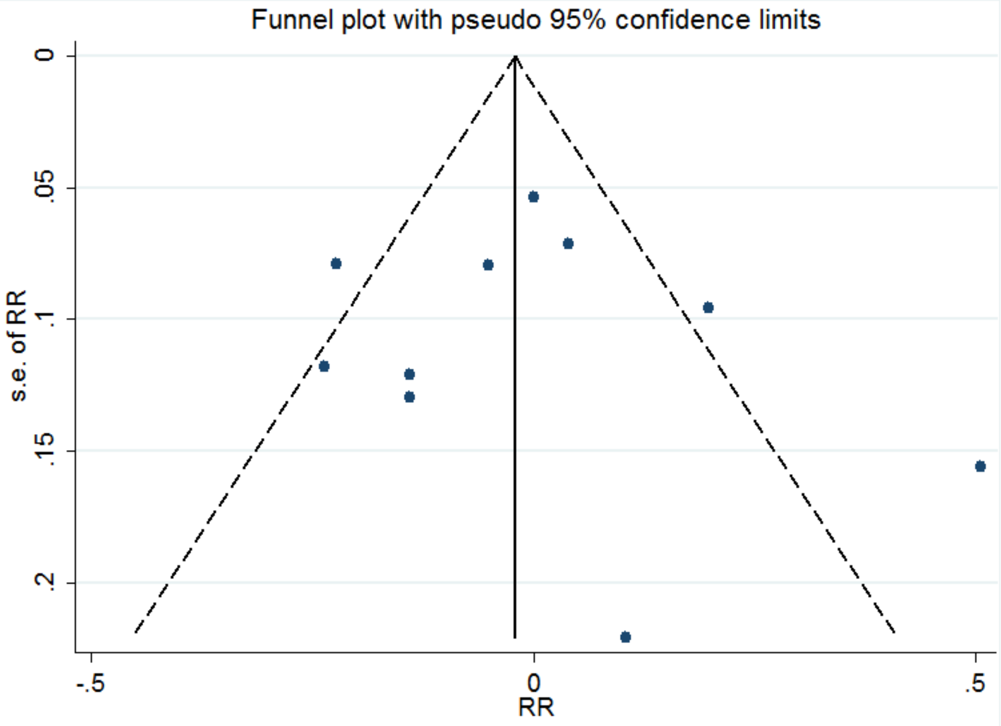

B CHD

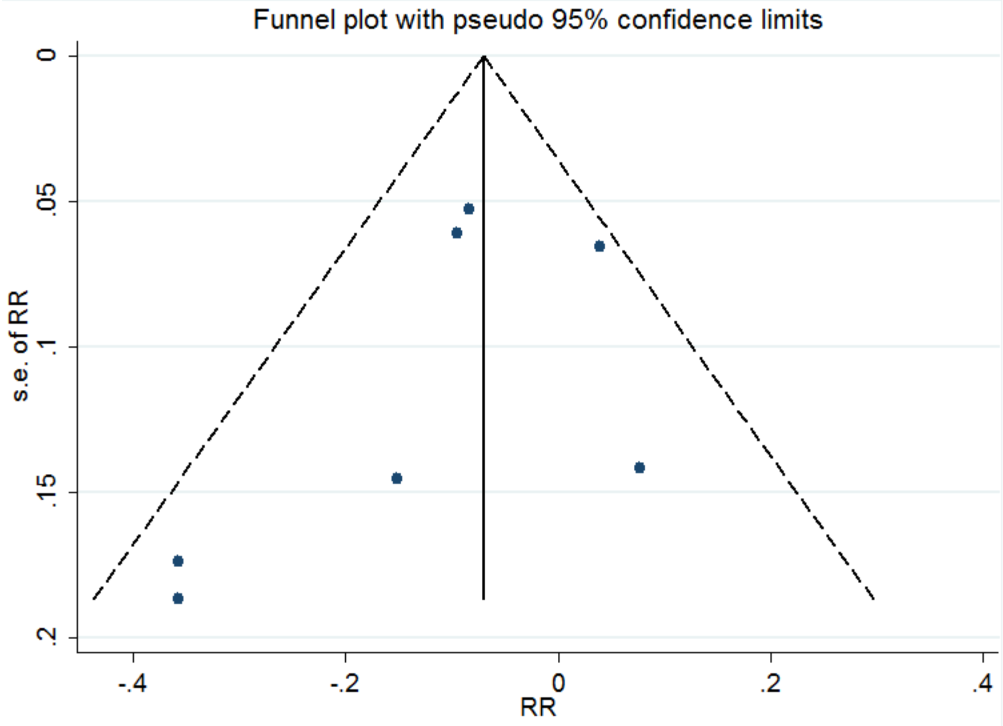

C Stroke

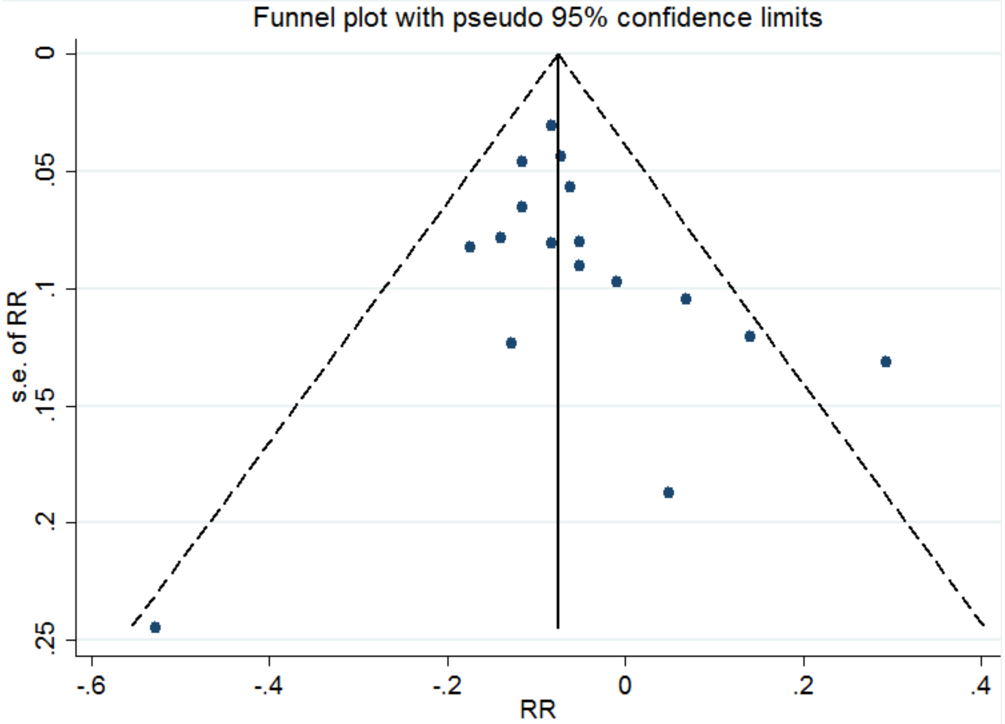

D T2DM

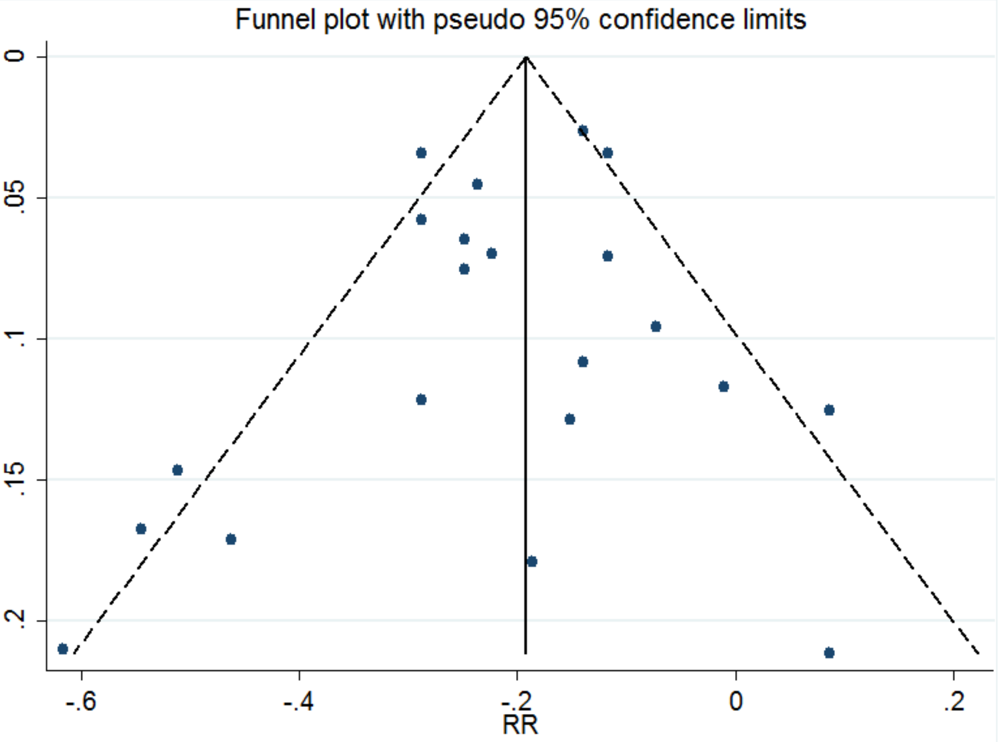

E All-cause mortality

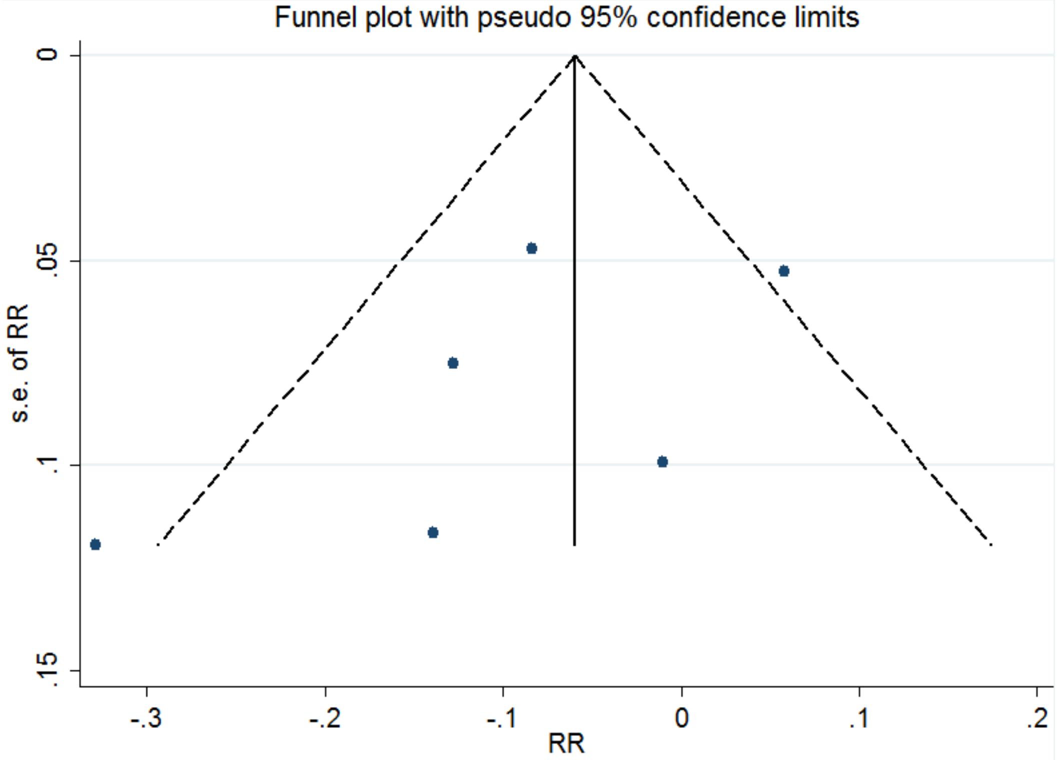

Supplement: Additional file 1: Table S1. — Quality assessment and references of all included prospective cohort studies. Table S2. Publication bias measured by Begg’s and Egger’s test. Figure S1. Funnel plots for studies of the association between the highest vs. lowest category in dietary magnesium intake and risk of total CVD (A), CHD (B), stroke (C), type 2 diabetes (D), and all-cause mortality (E). Figure S2. Funnel plots for studies of the association between per 100 mg/day increase in dietary magnesium intake and risk of total CVD (A), CHD (B), stroke (C), type 2 diabetes (D), and all-cause mortality (E). (PDF 1552 kb) [file 12916_2016_742_MOESM1_ESM.pdf]
